# Evaluation of average travel delay caused by moving bottlenecks on highways

**DOI:** 10.1371/journal.pone.0183442

**Published:** 2017-08-30

**Authors:** Xueyan Wei, Chengcheng Xu, Wei Wang, Menglin Yang, Xiaoma Ren

**Affiliations:** 1 Jiangsu Key Laboratory of Urban ITS, Southeast University, Nanjing, Jiangsu, China; 2 Jiangsu Province Collaborative Innovation Center of Modern Urban Traffic Technologies, Southeast University, Nanjing, Jiangsu, China; 3 Department of Urban Traffic, Hisense TransTech Co., Ltd., Qingdao, Shandong, China; Beihang University, CHINA

## Abstract

This paper presents a modelling framework to evaluate travel delay of all vehicles influenced by moving bottlenecks on highways. During the derivation of analytical formulas, the arrival of slow vehicles was approximated by a Poisson process based on the assumption that they occupied a constant low proportion of the traffic stream. The mathematical analysis process was developed from moving bottlenecks with the same velocity to those with multiple different velocities, and the closed-form expression of expected average travel delay was obtained by utilizing kinematic-wave moving bottleneck theory, gap acceptance theory, probability theory and renewal theory. Model validation and parameters sensitive analysis were conducted by simulation relying on the open source database of US highway 10. The maximum passing rate and the macroscopic parameters of initial traffic state with maximum delay could be found by means of approximate formulas. The proposed modeling framework can be applied for evaluating impacts of slow vehicles on highway operation quantifiably, based on which traffic managements like truck prohibited period decision and speed or lane restriction could be made more scientifically.

## Introduction

In mixed-traffic flow, the presence of a slow vehicle (SV) may cause a bottleneck for traffic stream with normal travel speed, which is moving with the running of that slow vehicle. SVs can be trucks, working vehicles and even cars driving by cautious drivers. These “moving bottlenecks (MBs)” make great contributions to the degradation of highway capacity and level of service, which has been confirmed by experimental findings [1]. Hence, it is indispensable to incorporate the influence mechanism of MBs into practical traffic models.

The existing studies on the influence mechanism of MBs can be classified into two categories. One is early studies on the exploration of characteristics of a MB and the other is subsequent studies on qualitative and quantitative influence of MBs on capacity.

Early studies focused on exploring characteristics of MBs. The concept of “moving bottlenecks (MBs)” describing the effect of bottlenecks caused by SVs was proposed by Gazis and Herman [2] firstly and was improved by Newell [3] who introduced the kinematic wave theory to analyze the influencing process of a MB on traffic stream theoretically. Later, Muñoz and Daganzo [1] diagnosed a MB from field data by adopting the oblique coordinate system [4] and the characteristics of a MB were then presented detailedly from field observation. Meanwhile, qualitative analysis of passing rates was conducted through two freeway experiments by them. However, all these studies only focus on one single MB regardless of possible interactions between two MBs. The interactions were firstly analyzed depending on SVs’ relative positions, which included arriving separately, arriving in pairs and blocking two lanes, and arriving in pairs and blocking one lane [5], with homogeneity assumption. Based on these almost complete theories of MBs, numerical analytical method of multiple MBs was introduced through modelling the time-space trajectories of MBs approximately by step functions with steps equal to the lattice spacing, and the interaction of two MBs traveling in the same lane with different speed was then firstly illustrated [6,7].

Afterwards, the effect of MBs on capacity became a prevailing research focus, which was first formulated by Laval [8] through analysis of simplified stochastic processes in one lane and was improved subsequently to multiple types of MBs in multilane [9]. As its extensive applications, Juran et al.[10] analyzed MBs at network level together with dynamic traffic assignment, and Liu [11] modified the CTM model, by using methods from the lagged CTM, to take MBs of buses into consideration by directly adopting the approximate formulas of [8], whereas all MBs were taken as a single MB without interactions in both studies. As its extensive research, Shiomi et al.[12] developed a model of platoon formation behind a bottleneck and a model of speed transitions within a platoon, based on which could get the conclusion that a regulation relating to the maximum and minimum speed limitation would reduce the occurrence of traffic breakdown and improve the efficiency of expressways. Also, it was found that effect of MBs on capacity became more remarkable when the coupling effect of multiple MBs occurred and that increasing the maximum speed of SVs could reduce the effect of MBs on capacity [13]. Accordingly, interactions between two MBs were analyzed by classifying them into three situations: no interaction, stop and go, and fully congested [14]. Similarly, a numerical method of a strongly coupled PDE-ODE system that described the MBs created by several buses with the same speed law revealed interactions with the shock wave and described time evolution of cars density and buses positions which illustrated transition from free flow to stop-and-go waves and reduction of distance between two following buses [15].

However, these previous researches did not take both passing rate and probabilities of occurrence of various interactions among MBs into account to evaluate the impacts of MBs on traffic operation. As for traffic information like traffic volume, travel time, travel speed and traffic condition, many researches without separately considering the influence of MBs on traffic operation had been conducted with considerable effectiveness [16–20]. But for travel delay caused by SVs mixed in traffic stream, MBs are dominant elements. Hence, when one evaluates average travel delay in terms of MBs, the ignorance of passing rate will magnify that of influenced vehicles, and it is improper to develop statistically significant average travel delay model while probabilities of occurrence of various interactions among MBs are neglected. Therefore, both of the two factors should be considered to provide more accurate advice for traffic managements.

This paper will propose a modeling framework based on kinematic wave theory to evaluate effects of MBs on average travel delay model. During the derivation of analytical formulas, passing rate and probabilities of occurrence of various interactions among MBs will be both taken into consideration. Particularly, the model will consider different cases of moving bottlenecks, including moving bottlenecks with a constant speed and multiple moving bottlenecks with constant and different speeds, by application of probability theory, which substantially improves the existing studies. The research results will promote a better understanding of impacts of MBs on traffic stream from statistical aspect and will help transportation professionals develop effective SVs restriction strategies to achieve better travel conditions.

The remainder of this paper is organized as follows. General framework dealing with MBs is presented in the first section. At the end of the first section, the framework of analytical process is given, based on which development of proposed model is demonstrated in subsequent two sections. The case of MBs with the same velocity is analyzed in the second section, and the third section presents case of MBs with different velocities. In the last section, model validation and parameters sensitive analysis are conducted. In addition, notations used during the derivation of the final approximate formula are tabulated in S1 File.

## General framework

The approach proposed in this paper is based on Newell’s kinematic wave theory of MB (KW-MB theory)[3], according to which the forming and discharging process of queue caused by a single MB can be illustrated in the diagram of flow-density curve and time-space plane. Generally, there are two different analysis methods depending on whether passing rate is considered.

### Without passing

**Fig 1:**
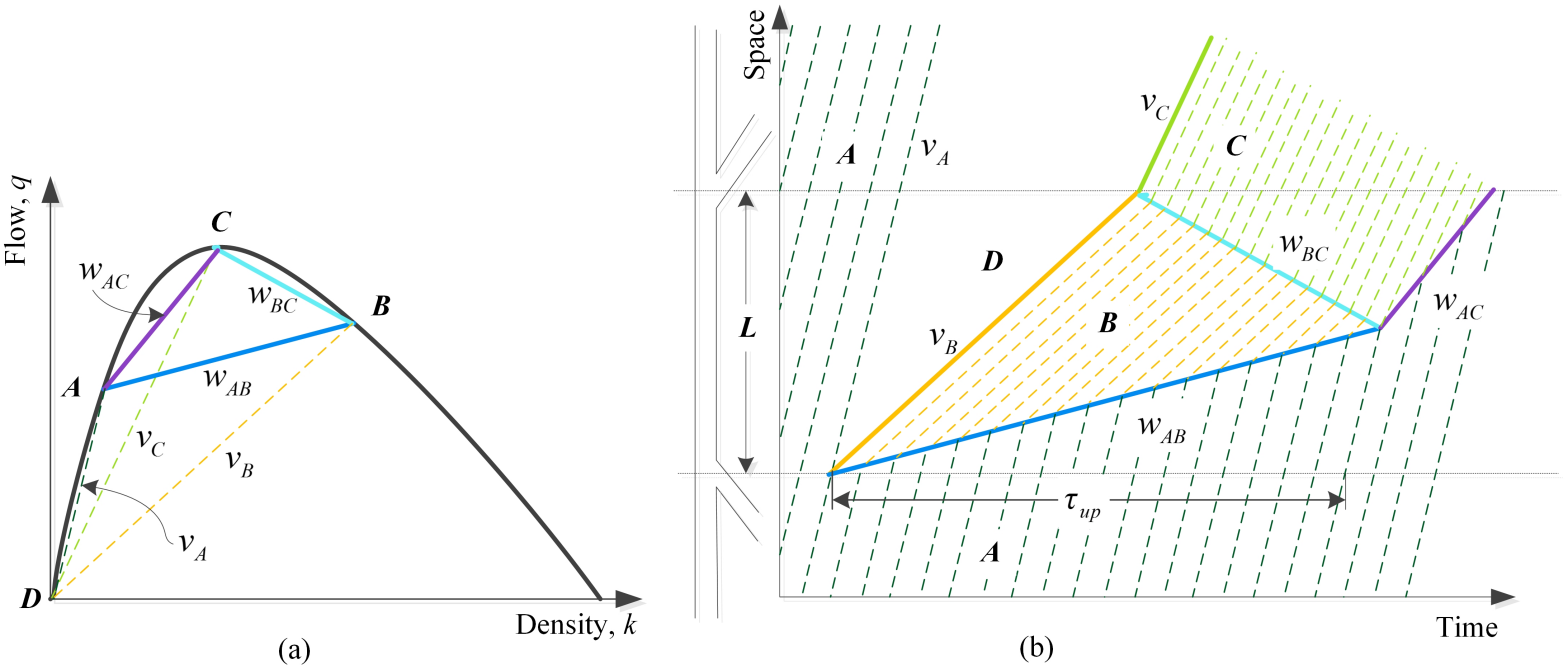
(a) Possible stationary states on the flow-density plane on a highway lane with a MB without passing; (b) the corresponding influencing process of a single MB in time-space plane.

The simplified case when no vehicle queuing upstream of the SV is assumed to change lanes and pass over it is shown in Fig 1. Point *A* represents the initial traffic state without influence of a MB, point *B* represents the traffic state of queue upstream of the SV which is defined as the traffic state of the MB, and point *C* represents the traffic state of capacity. In agreement with early researches [3,9], vehicles queuing upstream of the MB are assumed to travel with the uniform velocity equal to *v*_*B*_ as a platoon. We set the time a SV enters a highway flowing at state *A* as zero time and the position as zero position. The time *τ*_*up*_ will be called the disturbance time, during which vehicles in state *A* supposed to arrive at zero position will be influenced by the MB and join the queue. Eq 1 gives the solving formula of *τ*_*up*_.

$$\tau_{up}=\frac{L\left(v_{A}-w_{AB}\right)(v_{B}-w_{BC})}{v_{A}v_{B}(w_{AB}-w_{BC})}$$ (1)

Where *L* is the travel distance of the SV, of which the segment may be an uphill grade, steeped downgrade, sharp horizontal curve or other stream of vehicles moving consistently., *v*_*A*_ and *v*_*B*_ are the travel velocity in traffic state *A* and B respectively, and *w*_*AB*_, *w*_*BC*_ and *w*_*AC*_ represent the propagation velocity of traffic shock wave caused by the transition between traffic states.

However, with regard to the more realistic situation, vehicles queuing upstream of a SV are more likely to change lanes and pass over it rather than follow the platoon whenever it is possible. Previous studies [1,4] were implemented to reveal the qualitative relationship among passing rate, travel speed of the SV, and downstream flow and density with a series of controlled experiments. Nevertheless, the quantitative relationship between passing rate and related factors remains to explore.

### With passing

In this paper the passing rate is quantified with two critical parameters, the critical gap of lane-changing and the follow-up time that cost by one more lane-changing maneuver together with former ones using the same gap, based on the gap acceptance theory. We introduce the symbol *Γ* as the critical gap and the parameter *η* as the follow-up time, and assume there exists no individual differences among drivers of vehicles influenced by the same MB to decide changing lanes or not, which means *Γ* and *η* are constant for a particular MB but variable for different MBs. In the field of lane-changing studying, critical gap and lane-changing duration had been obtained by observation and statistics or modeling analysis methods [21].

**Fig 2:**
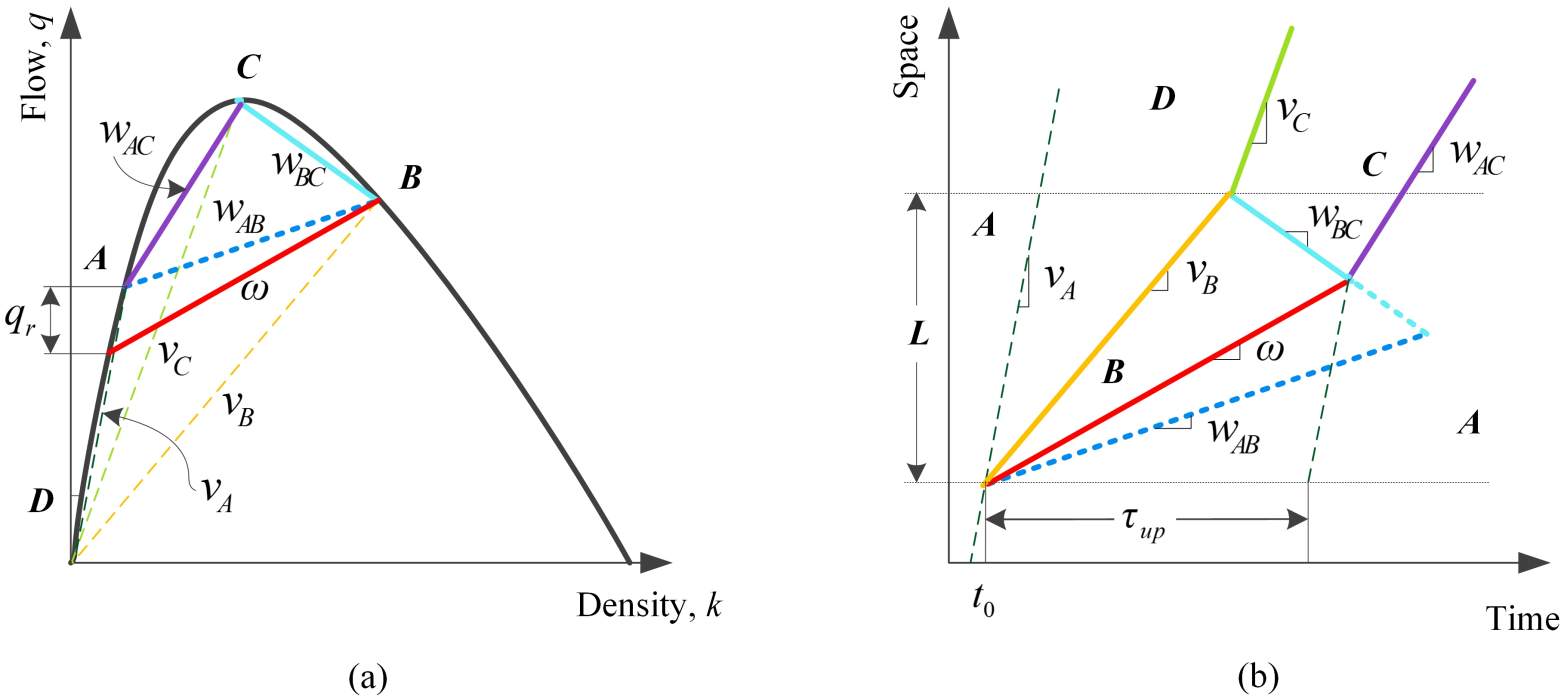
(a) Possible stationary states on the flow-density plane on a highway lane with a MB with passing; (b) the influencing process of a single MB with passing compared to that without passing in time-space plane.

As shown in Fig 2(a), traffic states of right lane and left lane are both in point *A* without MBs disturbance. Once a MB appears, queue begins to form upstream of the MB and vehicles in right lane influenced by it will change to the left lane using acceptable gaps. In the left adjacent lane, the arrival rate of vehicles in state *A* is *λ*_*A*_ = *q*_A_, and the average headway equals 1/*λ*_*A*_. According to gap acceptance theory, if one knows the headway distribution of state *A*, the expected value of admissible changing lane vehicle number using a headway in left lane can be calculated. Here we give Eq 2 by assuming a Poisson distribution for example.

$$E\left(m\right)=\underset{n\to +\infty }{\text{lim}}\left(\sum_{i=0}^{n-1}e^{-\lambda_{A}\left(\Gamma +i\eta \right)}-ne^{-\lambda_{A}\left(\Gamma +n\eta \right)}\right)=\frac{e^{-\lambda_{A}\Gamma }}{1-e^{-\lambda_{A}\eta }}$$ (2)

Therefore, the maximum passing rate *q*_*r*_ can be achieved by

$$q_{r}=\lambda_{A}E\left(m\right)$$ (3)

Upstream of the MB, the arrival rate of vehicles influenced by it, represented by *λ*_*q*_, can be computed by

$$\lambda_{q}=\lambda_{A}\left(1-w_{AB}/v_{A}\right)$$ (4)

If *λ*_*q*_*≤ q*_*r*_, all influenced vehicles would take advantage of acceptable gaps to change lanes and achieve better travel conditions, in other words, *q*_*r*_ equals *λ*_*q*_. Otherwise, redundant vehicles have to follow with it and the queue will propagate towards upstream with a shock wave velocity *ω* formulated as Eq 5, which is the situation studied in this paper.

$$\omega =\frac{\Delta q}{\Delta k}=\frac{q_{B}-\left(\lambda_{q}-q_{r}\right)}{k_{B}-k_{A}}$$ (5)

Furthermore, when taking passing rate into consideration, the formula of *τ*_*up*_ should be updated as

$$\tau_{up}=\frac{L\left(v_{A}-\omega \right)\left(v_{B}-w_{BC}\right)}{v_{A}v_{B}\left(\omega -w_{BC}\right)}$$ (6)

**Fig 3:**
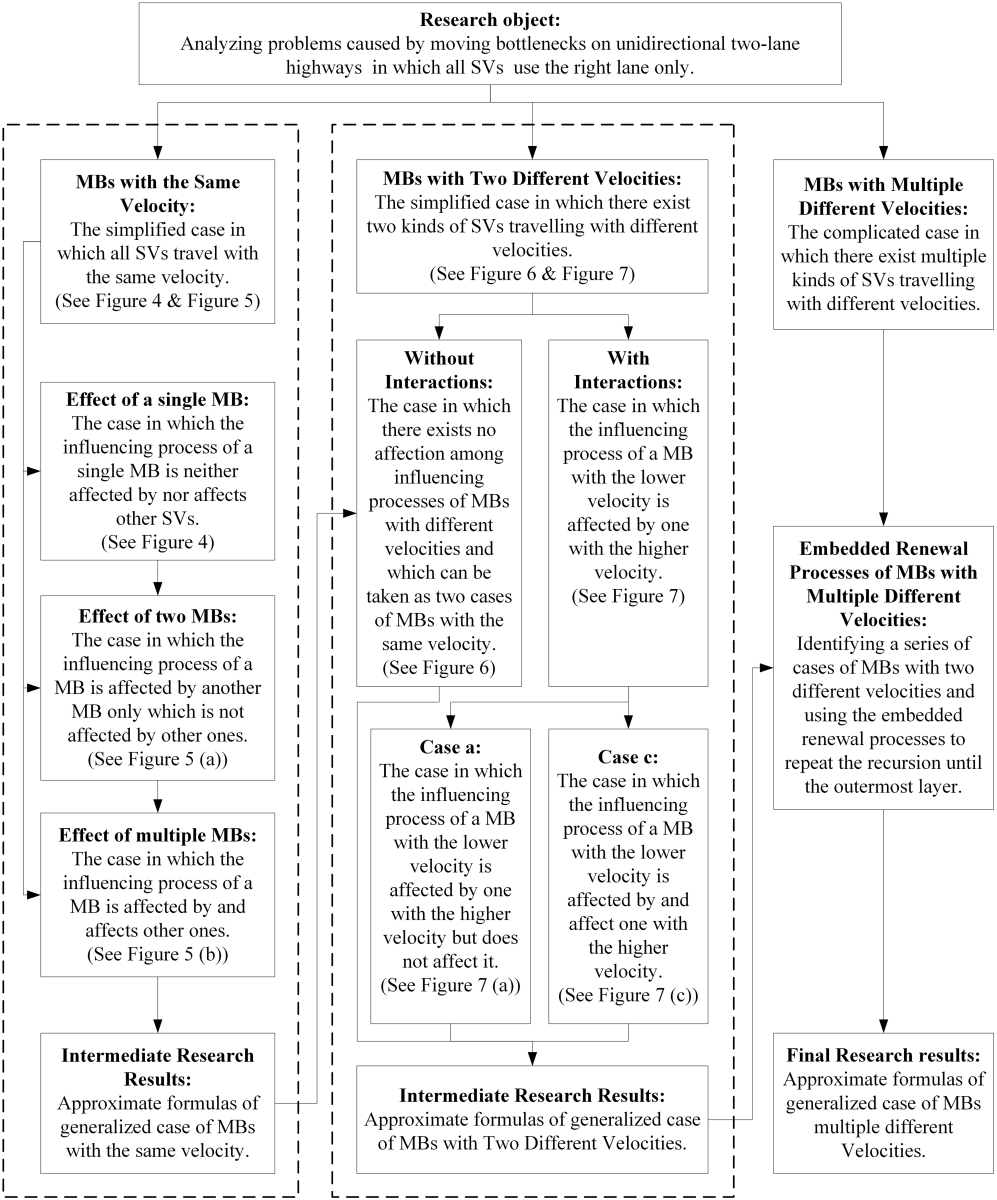
The analytical process for problems caused by moving bottlenecks on highways.

Based on the formula of passing rate, lengths of queues upstream of a MB and the ensuing vehicles delays can be formulated exactly with commonality and be predicted with good accuracy. According to the velocity of MBs, we solve the problem from MBs with the same velocity and then generalize to MBs with different velocities. The analytical process for problems caused by moving bottlenecks on highways is shown in Fig 3.

## MBs with the same velocity

In the situation when passing is available, we assume SVs only use the right lane and vehicles upstream of the MB only regard the left adjacent lane as the target lane of lane-changing behaviors throughout this paper, which can be seen as the single-lane case in [9]. In this section, effect of MBs with the same velocity are decomposed into three progressive cases to analyze. In the first subsection, formulations for the real-time length of queue *I*(*t*), the real-time position of a vehicle along with the queue *x*_*i*_(*t*) and the average travel delay of all vehicles influenced by a single MB D0 are derived. Due to the length limitation of this paper, we only present the formulations of the average travel delay in residual sections. As for other parameters, the modeling framework is still effective, and one just needs to give the formulations of them in each case while the probability of occurrence of all defined events sets are consistent and can be adopted directly.

### Effect of a single MB

Based on the headway of SVs, denoted by $\tilde{h}$, we conduct the research in two situations. If $\tilde{h}>\tau_{up}$, the influencing process of two MBs goes on respectively without an interaction, relying on which we can regard each MB as a single MB.

**Fig 4:**
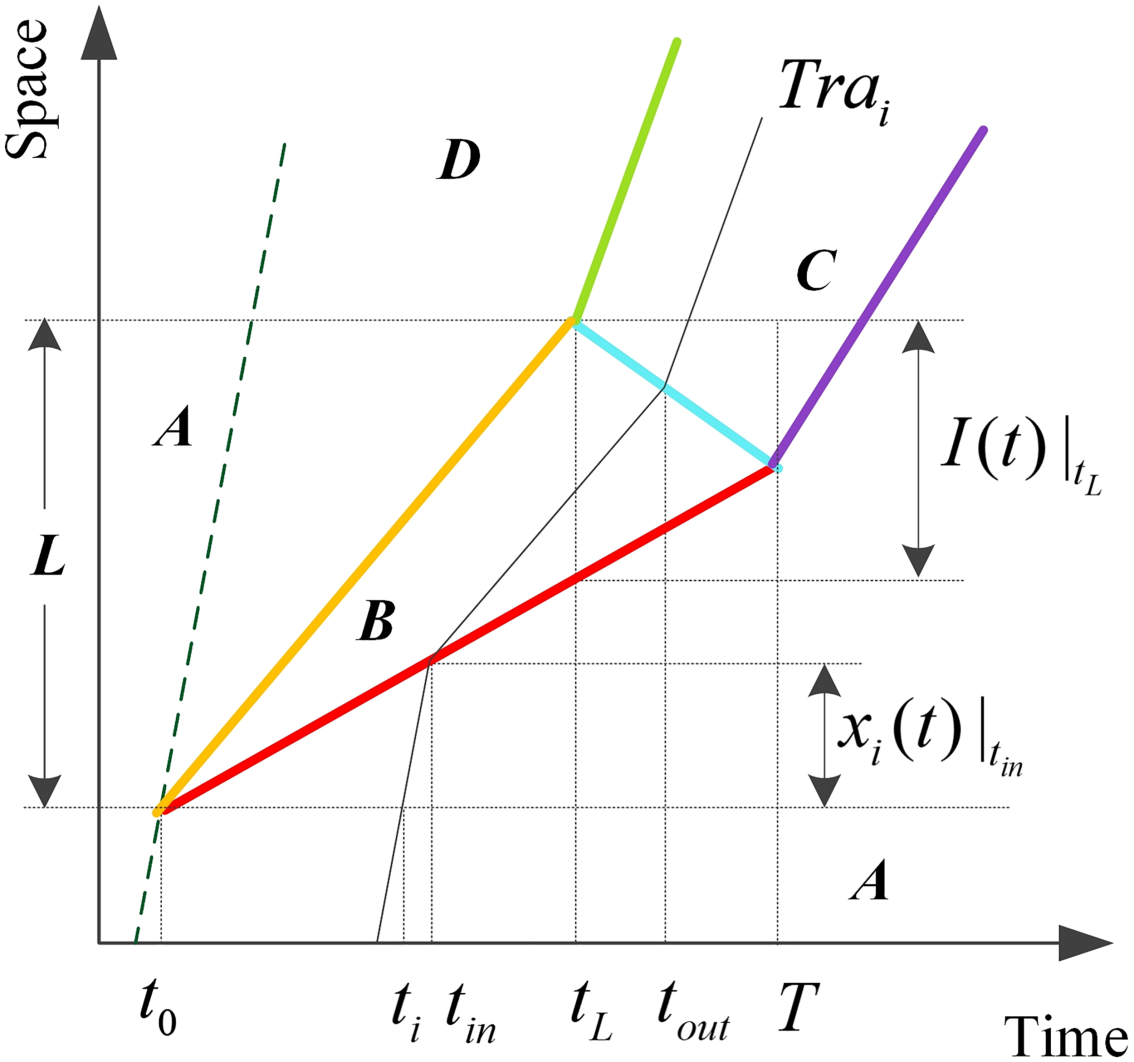
The analytic diagram of problems caused by a single MB in time-space plane.

As illustrated in Fig 4, *t*_*0*_ represents zero time and *t*_*L*_ expresses existence time of the MB while the queue clearance time is denoted by *T*. We introduce *φ* as the total number of vehicles influenced by the MB and joining the queue without passing opportunities within *T*, which can be estimated by

$$\varphi^{0}=\tau_{up}\left(q_{A}-q_{r}\right)$$ (7)

Where the superscript 0 indicates the case of a single MB.

Those vehicles in queue discharge gradually after the SV leaving the highway. Between *t*_*0*_ and *T*, the real-time length of queue upstream of the MB can be obtained by

$$Ι(t)=\{\begin{array}{ll} \left(v_{B}-\omega \right)t & t\leq t_{L}\\ \left(v_{B}-\omega \right)t_{L}+\left(w_{BC}-\omega \right)\left(t-t_{L}\right) & t_{L}<t<T\\ 0 & t\geq T \end{array}$$ (8a)

Where *T* and *t*_*L*_ can be calculated by

$$T=\frac{L-w_{BC}t_{L}}{\omega -w_{BC}},t_{L}=\frac{L}{v_{B}}$$ (8b)

A vehicle with a sequence number *i* supposed to arrive at zero position at *t*_*i*_ and later on joining the queue without passing opportunities within *T* will travel following the trajectory of *Tra*_*i*_ shown in Fig 4, and its real-time position can be achieved by

$$x_{i}(t)=\{\begin{array}{ll} v_{A}\left(t-t_{i}\right) & t\leq t_{in}\\ \omega \left(t_{in}-t_{i}\right)+v_{B}\left(t-t_{in}\right) & t_{in}<t\leq t_{out}\\ \omega t_{in}+v_{B}\left(t_{out}-t_{in}\right) & t_{out}<t \end{array}$$ (9a)

Where *t*_*in*_ represents the time at which vehicle *i* joins the queue and *t*_*out*_ represents that at which it discharges from the queue. The formulas of *t*_*i*_, t_in_ and t_out_ are given by

$$t_{i}=\frac{i}{q_{A}-q_{r}},t_{in}=\frac{v_{A}t_{i}}{v_{A}-\omega },t_{out}=\frac{L}{v_{B}}+\frac{v_{A}t_{i}\left(v_{B}-\omega \right)}{\left(v_{B}-w_{BC}\right)\left(v_{A}-\omega \right)}$$ (9b)

Notice that *i* ϵ *N*_+_, *i* ϵ [1, *φ*], and *t*_*i*_*≤τ*_*up*_.

The actual travel time of Vehicle *i* through distance *L* equals *t*_*Li*_ given by Eq 10 rather than *L*/*v*_*A*_, which indicates there exists travel delay of vehicle *i* resulting from the influence of the MB.

tLi=LvB+iqC−ti (10)

Therefore, the average travel delay of all vehicles influenced by a single MB can be estimated by

$$D^{0}=\frac{1}{\varphi^{0}+\tau_{up}q_{r}}\sum_{i=1}^{\varphi^{0}}\left(L\left(\frac{1}{v_{B}}-\frac{1}{v_{A}}\right)+i\left(\frac{1}{q_{C}}-\frac{1}{q_{A}-q_{r}}\right)\right)$$ (11)

### Effect of two MBs with the same velocity

**Fig 5:**
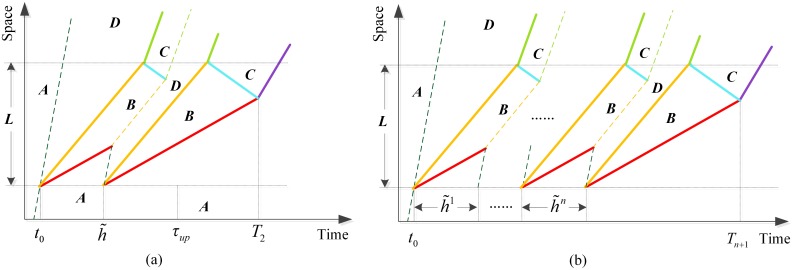
(a) The influence process of two MBs with the same velocity in time-space plane; (b) the influence process of multiple MBs with the same velocity in time-space plane.

On the contrary, as illustrated in Fig 5(a), vehicles arriving at zero position after $\tilde{h}$ will be influenced only by the second MB and follow the queue or change lanes when possible, while vehicles influenced by the first MB will have no passing opportunities after the last one joining the queue because of the fact that all passing opportunities in the left adjacent lane coming from upstream have been taken by vehicles influenced by the second MB.

In consideration of two MBs with the same velocity, the total number of vehicles influenced by them and joining the queue without passing opportunities within *T*_*2*_ is given by Eq 12a, and the formula of *t*_*Li*_ presents to be Eq 12b.

$$\varphi^{1}=(\tau_{up}+\tilde{h})(q_{A}-q_{r})$$ (12a)

tLi=LvB+i−h˜(qA−qr)Ξ(ti,h˜)qC+h˜Ξ(ti,h˜)−ti (12b)

Where the superscript 1 indicates the case of two MBs with the same velocity, and the variable $\Xi \left(t_{i},\tilde{h}\right)$ represents a 0–1 function whose return value only equals 0 or 1 based on a logical judgment and is formulated by

$$\Xi \left(t_{i},\tilde{h}\right)=\{\begin{array}{cc} 1 & t_{i}\geq \tilde{h}\\ 0 & t_{i}<\tilde{h} \end{array}$$ (12c)

With all above, the average travel delay of all vehicles influenced by two MBs with the same velocity can be estimated by

$$D^{1}=\frac{1}{\varphi^{1}+\left(\tau_{up}+\tilde{h}\right)q_{r}}\sum_{i=1}^{\varphi^{1}}\left(L\left(\frac{1}{v_{B}}-\frac{1}{v_{A}}\right)+i\left(\frac{1}{q_{C}}-\frac{1}{q_{A}-q_{r}}\right)+\tilde{h}\Xi \left(t_{i},\tilde{h}\right)\left(1-\frac{q_{A}-q_{r}}{q_{C}}\right)\right)$$ (12d)

### Effect of multiple MBs with the same velocity

When there exist multiple MBs, say the number is *n*+1 so that there exist *n* headways needing to be taken into consideration, disturbing the traffic stream with headway following a Poisson distribution, as illustrated in Fig 5(b), all of n headways are smaller than *τ*_*up*_, otherwise we deem them as several cases of a single MB or cases of two MBs or cases of multiple MBs separately. The total number of vehicles influenced by multiple MBs with the same velocity is given by Eq 13a and the average travel delay of all vehicles influenced by them can be estimated by Eq 13b.

$$\varphi^{n}=\left(\tau_{up}+\sum_{j=1}^{n}{\tilde{h}}^{j}\right)\left(q_{A}-q_{r}\right)$$ (13a)

$$D^{n}=\frac{1}{q_{A}\left(\tau_{up}+\sum_{j=1}^{n}{\tilde{h}}^{j}\right)}\sum_{i=1}^{\varphi^{n}}\left(L\left(\frac{1}{v_{B}}-\frac{1}{v_{A}}\right)+i\left(\frac{1}{q_{C}}-\frac{1}{q_{A}-q_{r}}\right)+\left(1-\frac{q_{A}-q_{r}}{q_{C}}\right)\sum_{k=1}^{n}{\tilde{h}}^{k}\Xi \left(t_{i},\sum_{j=1}^{k}{\tilde{h}}^{j}\right)\right)$$ (13b)

Where the superscript *n* indicates the case of (n+1) MBs with the same velocity, and the formula of variable $\Xi \left(t_{i},\tilde{h}\right)$ is given by

$$\Xi \left(t_{i},\tilde{h}\right)=\{\begin{array}{cc} 1 & t_{i}\geq \sum_{j=1}^{k}{\tilde{h}}^{j}\\ 0 & t_{i}<\sum_{j=1}^{k}{\tilde{h}}^{j} \end{array}$$ (13c)

We use *λ*_*SV*_ as the arrival rate of SVs which equals *p*_*SV*_*p*_*A*_. The probability density function of $\tilde{h}$ is shown to be $f\left(\tilde{h}\right)=\lambda_{SV}e^{-\lambda_{SV}\tilde{h}}$. The minimum safety headway between two SVs is denoted by $\hat{\tau }$. The expected value of $\tilde{h}$ between $\hat{\tau }$ and *τ*_*up*_ turns out to be

$$E\left(\tilde{h}\right){|}_{\hat{\tau }}^{\tau_{up}}=\int_{\hat{\tau }}^{\tau_{up}}\tilde{h}\frac{f\left(\tilde{h}\right)}{\int_{\hat{\tau }}^{\tau_{up}}f\left(\tilde{h}\right)}d\tilde{h}=\left(\hat{\tau }+\frac{1}{\lambda_{SV}}\right)\frac{e^{-\lambda_{SV}\hat{\tau }}}{e^{-\lambda_{SV}\hat{\tau }}-e^{-\lambda_{SV}\tau_{up}}}-\left(\tau_{up}+\frac{1}{\lambda_{SV}}\right)\frac{e^{-\lambda_{SV}\tau_{up}}}{e^{-\lambda_{SV}\hat{\tau }}-e^{-\lambda_{SV}\tau_{up}}}$$ (14a)

Then Eqs 13a and 13b simplify to

$$\varphi^{n}=\left(\tau_{up}+nE\left(\tilde{h}\right){|}_{\hat{\tau }}^{\tau_{up}}\right)\left(q_{A}-q_{r}\right)$$ (14b)

$$D^{n}=\frac{q_{A}-q_{r}}{q_{A}}\left(\begin{array}{l} L\left(\frac{1}{v_{B}}-\frac{1}{v_{A}}\right)+\left(\frac{1}{q_{C}}-\frac{1}{q_{A}-q_{r}}\right)\frac{\varphi^{n}+1}{2}\\ +\frac{nE\left(\tilde{h}\right){|}_{\hat{\tau }}^{\tau_{up}}}{\tau_{up}+nE\left(\tilde{h}\right){|}_{\hat{\tau }}^{\tau_{up}}}\left(\frac{\left(n-1\right)E\left(\tilde{h}\right){|}_{\hat{\tau }}^{\tau_{up}}}{2}+\tau_{up}\right)\left(1-\frac{q_{A}-q_{r}}{q_{C}}\right) \end{array}\right)$$ (14c)

The values of ${\tilde{h}}^{j}$, the headway between two MBs with the sequence number *j* and (*j*+1), conform to the concept of *n*-fold Bernoulli trials. The probability of ${\tilde{h}}^{j}<\tau_{up}$ equals ($1-e^{-\lambda_{SV}*\tau_{up}}$) while the probability of ${\tilde{h}}^{j}>\tau_{up}$ equals $e^{-\lambda_{SV}*\tau_{up}}$. Naturally, the probability of occurrence of influencing process of multiple MBs with the same velocity follows a geometric distribution with parameter $e^{-\lambda_{SV}*\tau_{up}}$ and therefore the probability of *D*^*n*^ turns out to be

$$F\left(D^{n}\right)=e^{-\lambda_{SV}\tau_{up}}{\left(1-e^{-\lambda_{SV}\tau_{up}}\right)}^{n}$$ (15)

Based on above analyses, we eventually draw a conclusion that the expected value of average travel delay of all vehicles influenced by all MBs with the same velocity *v*_*B*_ can be estimated by

$$E\left(D\right)=\sum_{i=0}^{+\infty }D^{i}F\left(D^{i}\right)$$ (16)

When it comes to the practical application, *i* in Eq 16 is determined based on the accuracy requirement.

## MBs with different velocities

We introduce subscript *j* to differentiate cases with different velocities, and *j∈N*_+_. As before, we adopt the convention that a higher *j*-subscript implies a higher speed. We assume there exist *N* kinds of MBs with a set of *N* discrete values *v*_*Bj*_ with cumulative distribution function $G\left(v_{Bj}\right)=\sum_{k=1}^{j}p_{SVk}$, *j* = 1…*N*, where *p*_*SVj*_ gives the proportion of SVs with velocity of *v*_*Bj*_. The passing rate *q*_*r*_ is a function of critical gap *Γ* and follow-up time *η* described as *q*_*r*_(*Γ*,*η*). In addition, *Γ* and *η* are functions of the velocity of MBs. Thus the passing rate of MBs with *v*_*Bj*_ is *q*_*rj*_(*Γ*_*j*_,*η*_*j*_). For ease of description, MB_*j*_s represent MBs with a velocity of *v*_*Bj*_. Then with regard to MB_*j*_s, Eq 16 can be generalized to

$$E\left(D_{j}\right)=\sum_{i=0}^{+\infty }D_{j}^{i}F\left(D_{j}^{i}\right)$$ (17a)

Where *E*(*D*_*j*_) represents the expected value of average travel delay of all vehicles influenced by MB_*j*_s only, *D*_*j*_^*i*^ whose formula is given by Eq 17b represents the average travel delay of vehicles influenced by (*i*+1) MB_*j*_s, and *F(D*_*j*_^*i*^*)* whose formula is given by Eq 17c represents the probability of *D*_*j*_^*i*^.

$$D_{j}^{i}=\frac{q_{A}-q_{rj}}{q_{A}}\left(\begin{array}{l} L\left(\frac{1}{v_{Bj}}-\frac{1}{v_{A}}\right)+\left(\frac{1}{q_{C}}-\frac{1}{q_{A}-q_{rj}}\right)\frac{\varphi_{j}^{i}+1}{2}\\ +\frac{iE\left(\tilde{h}\right){|}_{\hat{\tau }}^{\tau_{upj}}}{\tau_{upj}+iE\left(\tilde{h}\right){|}_{\hat{\tau }}^{\tau_{upj}}}\left(\frac{\left(i-1\right)E\left(\tilde{h}\right){|}_{\hat{\tau }}^{\tau_{upj}}}{2}+\tau_{upj}\right)\left(1-\frac{q_{A}-q_{rj}}{q_{C}}\right) \end{array}\right)$$ (17b)

$$F\left(D_{j}^{i}\right)=e^{-p_{SVj}\lambda_{SV}\tau_{upj}}{\left(1-e^{-p_{SVj}\lambda_{SV}\tau_{upj}}\right)}^{i}$$ (17c)

Notice that *φ*_*j*_^*i*^ represents the total number of vehicles influenced by (*i*+1) MB_*j*_s and joining the queue without passing opportunities, which is updated by

$$\varphi_{j}^{i}=\left(\tau_{upj}+iE\left(\tilde{h}\right){|}_{\hat{\tau }}^{\tau_{upj}}\right)\left(q_{A}-q_{rj}\right)$$ (17d)

Where the expected value of $\tilde{h}$ between $\hat{\tau }$ and *τ*_*upj*_ should be correspondingly updated by

$$E\left(\tilde{h}\right){|}_{\hat{\tau }}^{\tau_{upj}}=\left(\hat{\tau }+\frac{1}{p_{SVj}\lambda_{SV}}\right)\frac{e^{-p_{SVj}\lambda_{SV}\hat{\tau }}}{e^{-p_{SVj}\lambda_{SV}\hat{\tau }}-e^{-p_{SVj}\lambda_{SV}\tau_{upj}}}-\left(\tau_{upj}+\frac{1}{p_{SVj}\lambda_{SV}}\right)\frac{e^{-p_{SVj}\lambda_{SV}\tau_{upj}}}{e^{-p_{SVj}\lambda_{SV}\hat{\tau }}-e^{-p_{SVj}\lambda_{SV}\tau_{upj}}}$$ (17e)

Furthermore, the generalized formulas of *ω*_*j*_, *τ*_*upj*_ and *q*_*rj*_ are given by

$$\omega_{j}=\frac{q_{Bj}-\left(\lambda_{A}\left(1-w_{AB_{j}}/v_{A}\right)-q_{rj}\right)}{k_{Bj}-k_{A}}$$ (17f)

$$\tau_{upj}=\frac{L\left(v_{A}-\omega_{j}\right)\left(v_{Bj}-w_{B_{j}C}\right)}{v_{A}v_{Bj}\left(\omega_{j}-w_{B_{j}C}\right)}$$ (17g)

$$q_{rj}=\lambda_{A}\frac{e^{-\lambda_{A}\Gamma_{j}}}{1-e^{-\lambda_{A}\eta_{j}}}$$ (17h)

Here we assume a SV with higher velocity does not change lanes but follow the queue upstream of the MB with a lower velocity and therefore it is forced to travel at the same lower velocity as long as it is queuing behind the MB with a lower velocity. Once it has left the queue, its travelling velocity speeds up to its original one immediately. Also note that the MB with a lower velocity arriving at zero position within *τ*_*up*_ of a MB with a higher velocity is not affected by it [9].

### Effect of MBs with two different velocities

This section presents formulas of average travel delay in the case of MBs with only two different velocities where *N* = 2 and the result will be generalized to MBs with multiple different velocities in the next section. The effect of MBs with two different velocities is analyzed in the next two subsections, which are case without interactions and case with interactions.

In the case of two kinds of MBs, MB_1_s and MB_2_s, the proportion of SV_1_s and SV_2_s in all SVs is *p*_*SV*1_ and *p*_*SV*2_ respectively where the sum of them equals 100%. We note events that there exists an interaction between two MB_1_s as events set A, events that there exists an interaction between a MB_1_ and a MB_2_ as events set B, events that there exists no interaction between a MB_1_ and a MB_2_ as events set $\bar{\text{B}}$, which is the complementary events set of events set B, and events that there exists an interaction between a MB_1_ and another MB despite whether this MB is a MB_1_ or not. Consequently, the probabilities of occurrence of events set A, B and C are computed by

$$P_{1}\left(A\right)=1-e^{-p_{SV1}\lambda_{SV}\tau_{up1}}$$ (18a)

$$P_{1}\left(C\right)=1-e^{-\sum_{k=1}^{N}p_{SVk}\lambda_{SV}\tau_{up1}}$$ (18b)

$$P_{1}\left(B\right)=P_{1}\left(C\right)-P_{1}\left(A\right)=e^{-p_{SV1}\lambda_{SV}\tau_{up1}}-e^{-\sum_{k=1}^{N}p_{SVk}\lambda_{SV}\tau_{up1}}$$ (18c)

In this section, we classify all vehicles influenced by all MB_1_s and MB_2_s into three categories, which are vehicles from events set $\bar{\text{B}}$, influenced by all MBs except for those MB_1_s and MB_2_s from events set B, vehicles from events set B_a_, influenced by those MB_1_s and MB_2_s from events set B, and vehicles from events set B_c_, influenced by those MB_1_s and MB_2_s from events set B. Detailed analysis of the process is as follows.

#### Without interactions between MBs with different velocities

**Fig 6:**
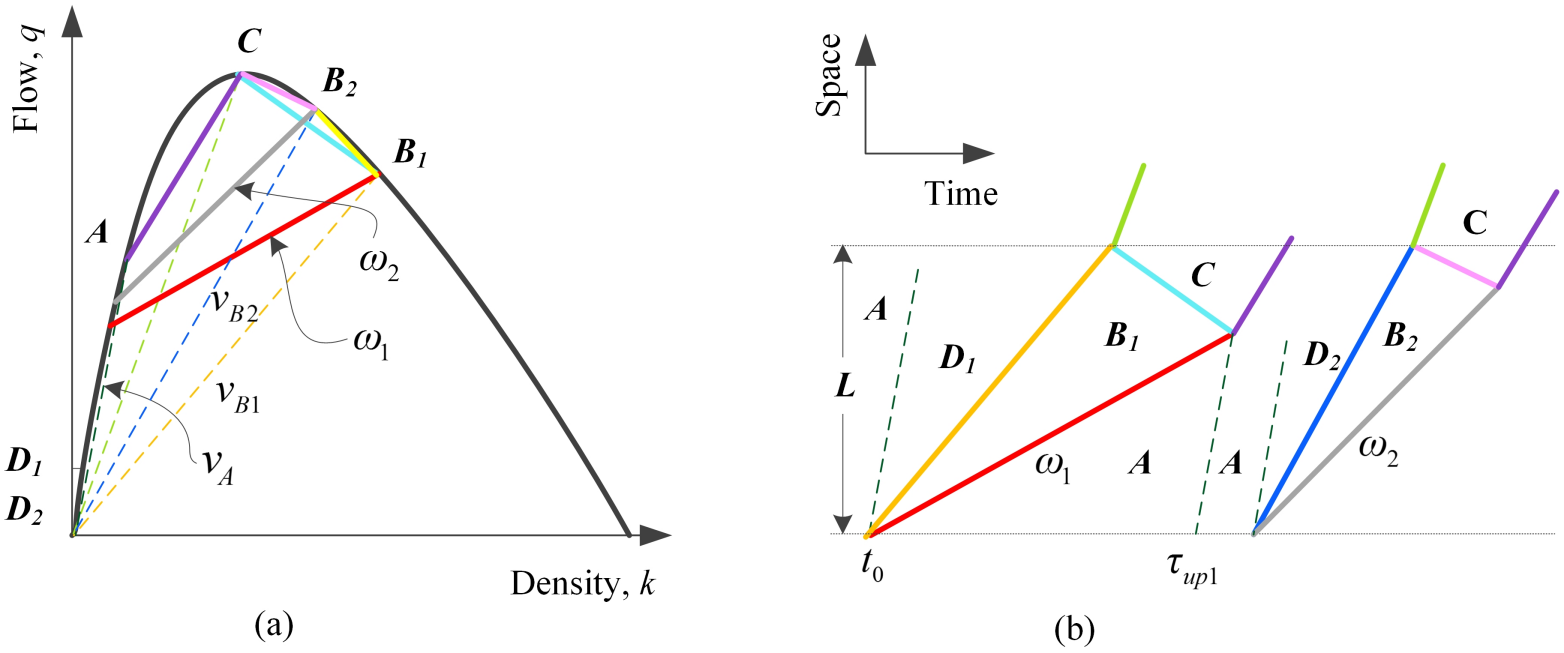
(a) Possible stationary states on the flow-density plane on a highway lane with MBs with two different velocities; (b) the influencing process of a MB_1_ and a MB_2_ without interactions in time-space plane.

If a MB_2_ arrived at zero position after *τ*_up1_, the MB_2_ would not affect the influencing process of the MB_1_ in front of it, seen in Fig 6, which constitutes events set $\bar{\text{B}}$. Hence, its probability of occurrence is given by

$$P_{1}\left(\bar{B}\right)=1+e^{-\sum_{k=1}^{N}p_{SVk}\lambda_{SV}\tau_{up1}}-e^{-p_{SV1}\lambda_{SV}\tau_{up1}}$$ (18d)

On account of no interactions, we can apply Eqs 17a–17h to MB_1_s and MB_2_s respectively to obtain *E*(*D*_1_) and *E*(*D*_2_). In this case, expected average travel delay of all vehicles influenced by MBs is given by

$$E\left(D_{1|2,\bar{B}}\right)=p_{SV1}E\left(D_{1}\right)+p_{SV2}E\left(D_{2}\right)$$ (19)

#### With interactions between MBs with different velocities

**Fig 7:**
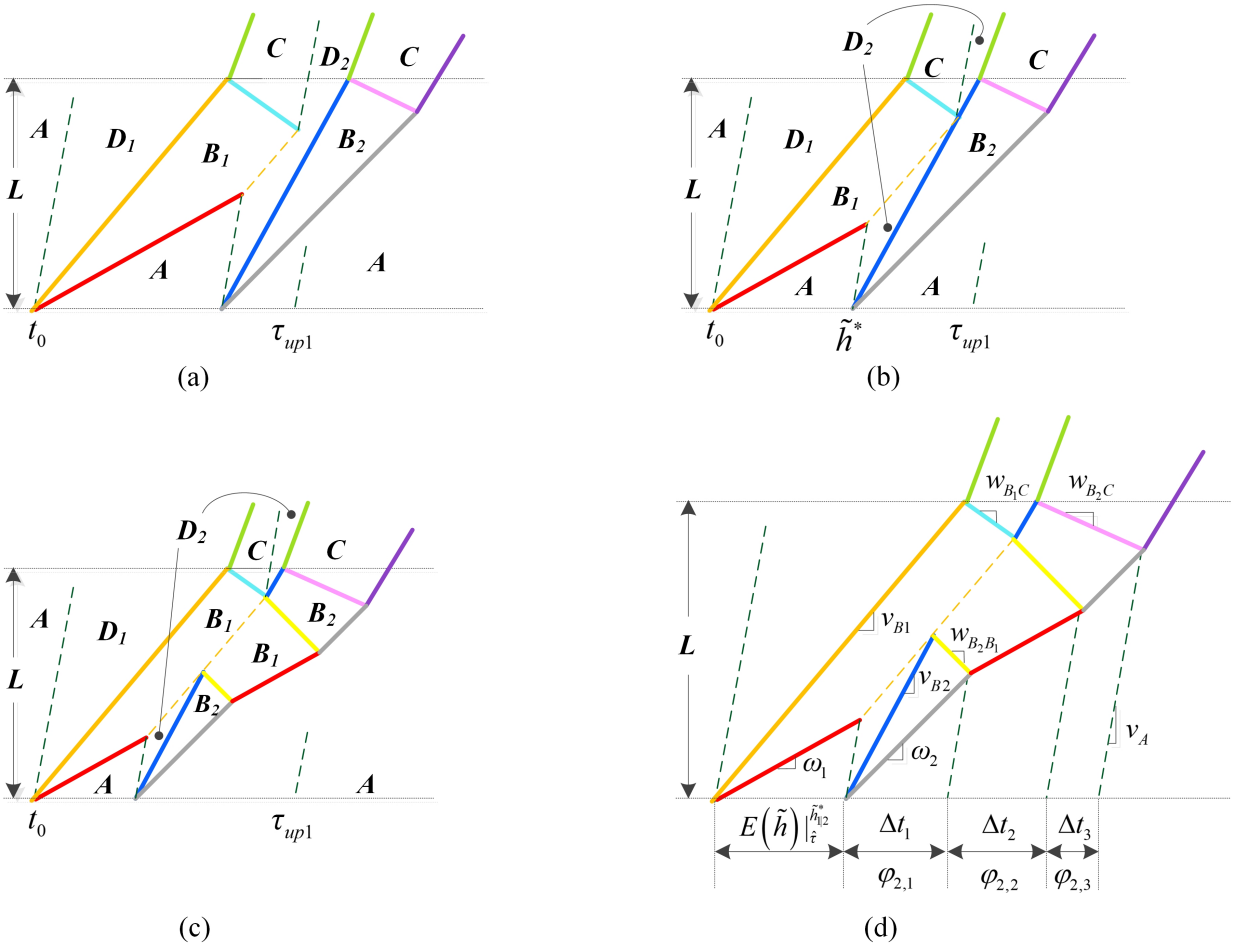
(a-c) The influencing process of a MB_1_ and a MB_2_ with an interaction in time-space plane; (d) an assistant diagram of (c) for analytical algorithm.

If a MB_2_ arrived at zero position within *τ*_up1_, the influencing process of it would affect the influencing process of the MB_1_ and be affected by it according to the headway between them, as shown in Fig 7(a)–7(c). The critical case is shown as Fig 7(b) when the headway between a MB_1_ and a MB_2_ equals ${\tilde{h}}_{1|2}^{*}$ which is given by

$${\tilde{h}}_{1|2}^{*}=\frac{L\left(v_{B2}-v_{B1}\right)\left(v_{A}-\omega_{1}\right)}{v_{B2}\omega_{1}\left(v_{A}-v_{B1}\right)}$$ (20a)

In the case shown as Fig 7(a) when the headway between a MB_1_ and a MB_2_ is larger than ${\tilde{h}}_{1|2}^{*}$, SV_2_s would not join the queue upstream of MB_1_s, which indicates the influencing process of a MB_1_ would be influenced by a MB_2_ but not affect it. Thus, the average delay of vehicles influenced by the single MB_2_ in this case can be obtained by Eq 11. The headway between a MB_1_ and a MB_2_ in this case lies within the range from ${\tilde{h}}_{1|2}^{*}$ to *τ*_*up*1_ with the expected value given by

$$E\left(\tilde{h}\right){|}_{{\tilde{h}}_{1|2}^{*}}^{\tau_{up1}}=\left({\tilde{h}}_{1|2}^{*}+\frac{1}{\sum_{k=j}^{N}p_{SVk}\lambda_{SV}}\right)\frac{e^{-\sum_{k=j}^{N}p_{SVk}\lambda_{SV}{\tilde{h}}_{1|2}^{*}}}{e^{-\sum_{k=j}^{N}p_{SVk}\lambda_{SV}{\tilde{h}}_{1|2}^{*}}-e^{-\sum_{k=j}^{N}p_{SVk}\lambda_{SV}\tau_{up1}}}-\left(\tau_{up1}+\frac{1}{\lambda_{SV}}\right)\frac{e^{-\sum_{k=j}^{N}p_{SVk}\lambda_{SV}\tau_{up1}}}{e^{-\sum_{k=j}^{N}p_{SVk}\lambda_{SV}{\tilde{h}}_{1|2}^{*}}-e^{-\sum_{k=j}^{N}p_{SVk}\lambda_{SV}\tau_{up1}}}$$ (20b)

Therefore, the total number of vehicles influenced by a MB_1_ without passing opportunities in this case, *φ*_1,*a*_, is refreshed to be Eq 20c while that by a MB_2_, *φ*_2,*a*_, is refreshed to be Eq 20d.

$$\varphi_{1,a}=E\left(\tilde{h}\right){|}_{{\tilde{h}}_{1|2}^{*}}^{\tau_{up1}}\left(q_{A}-q_{r1}\right)$$ (20c)

$$\varphi_{2,a}=\tau_{up2}\left(q_{A}-q_{r2}\right)$$ (20d)

The expected average delay of all vehicles influenced by MB_1_s and MB_2_s in case (a), *E*(*D*_1|2,*a*_), turns out to be

$$E\left(D_{1|2,a}\right)=\frac{1}{q_{A}\left(E\left(\tilde{h}\right){|}_{{\tilde{h}}_{1|2}^{*}}^{\tau_{up1}}+\tau_{up2}\right)}\left(\begin{array}{l} \varphi_{1,a}L\left(\frac{1}{v_{B1}}-\frac{1}{v_{A}}\right)+\frac{\varphi_{1,a}\left(\varphi_{1,a}+1\right)}{2}\left(\frac{1}{q_{C}}-\frac{1}{q_{A}-q_{r1}}\right)\\ +\varphi_{2,a}L\left(\frac{1}{v_{B2}}-\frac{1}{v_{A}}\right)+\frac{\varphi_{2,a}\left(\varphi_{2,a}+1\right)}{2}\left(\frac{1}{q_{C}}-\frac{1}{q_{A}-q_{r2}}\right) \end{array}\right)$$ (20e)

We note the case of Fig 7(a) as a subset of events set B, described as events set B_a_. Its probability of occurrence, *P*_1_(*B*_*a*_), equals that of the headway of SVs including all SV_1_s and all SV_2_s lying within the range between ${\tilde{h}}_{1|2}^{*}$ and *τ*_*up*1_ minus that of the headway of all SV_1_s lying within the same range, which is given by

$$P_{1}\left(B_{a}\right)=\left(1-e^{-\sum_{k=j}^{N}p_{SVk}\lambda_{SV}\tau_{up1}}\right)e^{-\sum_{k=j}^{N}p_{SVk}\lambda_{SV}{\tilde{h}}_{1|2}^{*}}-\left(1-e^{-p_{SV1}\lambda_{SV}\tau_{up1}}\right)e^{-p_{SV1}\lambda_{SV}{\tilde{h}}_{1|2}^{*}}$$ (21)

In addition, we note the other case of Fig 7(c) as another subset of events set B, described as events set B_c_. Consequently, its probability of occurrence, *P*_1_(*B*_*c*_), turns out to be

$$P_{1}\left(B_{c}\right)=P_{1}\left(B\right)-P_{1}\left(B_{a}\right)$$ (22)

In the case shown as Fig 7(c) when the headway between a MB_1_ and a MB_2_ is within ${\tilde{h}}_{1|2}^{*}$, we divide the influencing process of a MB_2_ into three stages according to the travel velocity after queuing, seen in Fig 7(d). *φ*_2,*ci*_ of vehicles arriving at zero position during Δ*t*_2,*ci*_ will queue without passing opportunities, where the subscript 2 represents the MB_2_ and *i* represents sequence number of stage, *i* = 1, 2 or 3. The headway between a MB_1_ and a MB_2_ in this case lies within the range from $\hat{\tau }$ to ${\tilde{h}}_{1|2}^{*}$ with the expected value given by

$$E\left(\tilde{h}\right){|}_{\hat{\tau }}^{{\tilde{h}}_{1|2}^{*}}=\left(\hat{\tau }+\frac{1}{\sum_{k=j}^{N}p_{SVk}\lambda_{SV}}\right)\frac{e^{-\sum_{k=j}^{N}p_{SVk}\lambda_{SV}\hat{\tau }}}{e^{-\sum_{k=j}^{N}p_{SVk}\lambda_{SV}\hat{\tau }}-e^{-\sum_{k=j}^{N}p_{SVk}\lambda_{SV}{\tilde{h}}_{1|2}^{*}}}-\left({\tilde{h}}_{1|2}^{*}+\frac{1}{\sum_{k=j}^{N}p_{SVk}\lambda_{SV}}\right)\frac{e^{-\sum_{k=j}^{N}p_{SVk}\lambda_{SV}{\tilde{h}}_{1|2}^{*}}}{e^{-\sum_{k=j}^{N}p_{SVk}\lambda_{SV}\hat{\tau }}-e^{-\sum_{k=j}^{N}p_{SVk}\lambda_{SV}{\tilde{h}}_{1|2}^{*}}}$$ (23)

The actual travel time *t*_*Li*_ of Vehicle *i* influenced by MB_2_s without passing opportunity through distance L in case (c) can be captured by Eqs 24a–24d:

tLi=LvB1+ω1E(h˜)|τ^h˜1|2*−vAE(h˜)|τ^h˜1|2*(vB2(ω1−wB1C)+wB1C(vB1−ω1))vB2(vB1−wB1C)+iqC−ti (24a)

$$t_{i}=\{\begin{array}{ll} \frac{i}{q_{A}-q_{r2}} & i\leq \varphi_{2,c1}\\ \frac{i-\varphi_{2,c1}}{q_{A}-q_{r1}} & \varphi_{2,c1}<i\leq \varphi_{2,c1}+\varphi_{2,c2}\\ \frac{i-\varphi_{2,c1}-\varphi_{2,c2}}{q_{A}-q_{r2}} & \varphi_{2,c1}+\varphi_{2,c2}<i\leq \varphi_{2,c} \end{array}$$ (24b)

$$\{\begin{array}{l} \varphi_{2,c1}=\left(q_{A}-q_{rj+1}\right)\Delta t_{2,c1}\\ \varphi_{2,c2}=\left(q_{A}-q_{rj}\right)\Delta t_{2,c2}\\ \varphi_{2,c3}=\left(q_{A}-q_{rj+1}\right)\Delta t_{2,c3}\\ \varphi_{2,c}=\varphi_{2,c1}+\varphi_{2,c2}+\varphi_{2,c3} \end{array}$$ (24c)

$$\varphi_{1,c}=E\left(\tilde{h}\right){|}_{\hat{\tau }}^{{\tilde{h}}_{1|2}^{*}}\left(q_{A}-q_{r1}\right)$$ (24d)

Where *φ*_2,*c*1_, *φ*_2,*c*2_ and *φ*_2,*c*3_ represent the number of vehicles influenced by MB_2_s in events set B_c_ at stage 1, 2 and 3, respectively, which lasts *Δt*_2,*c*1_, *Δt*_2,*c*2_ and *Δt*_2,*c*3_, respectively. The values of *Δt*_2,*c*1_, *Δt*_2,*c*2_ and *Δt*_2,*c*3_ can easily be obtained through the geometrical relationships shown in Fig 7(d). And *φ*_1,*c*_ and *φ*_2,*c*_ represent the total number of vehicles influenced by MB_1_s and MB_2_s respectively in events set B_c_.

For simplicity, we introduce a constant *C*_1|2_ to describe the common elements of those vehicles, and the value of the constant *C*_1|2_ is given by

C1|2=LvB1+ω1E(h˜)|τ^h˜1|2*−vAE(h˜)|τ^h˜1|2*(vB2(ω1−wB1C)+wB1C(vB1−ω1))vB2(vB1−wB1C)−LvA (24e)

Hence, the average travel delay of all vehicles influenced by MB_1_s and MB_2_s in Fig 7(c) can be estimated by

$$\begin{array}{c} E\left(D_{1|2,c}\right)=\frac{1}{q_{A}\left(E\left(\tilde{h}\right){|}_{\hat{\tau }}^{{\tilde{h}}_{1|2}^{*}}+\Delta t_{2,c1}+\Delta t_{2,c2}+\Delta t_{2,c3}\right)}\\ {}*\left(\begin{array}{l} \varphi_{1,c}L\left(\frac{1}{v_{B1}}-\frac{1}{v_{A}}\right)+\frac{\varphi_{1,c}\left(\varphi_{1,c}+1\right)}{2}\left(\frac{1}{q_{C}}-\frac{1}{q_{A}-q_{r1}}\right)+\varphi_{2,c}\left(C_{1|2}+\frac{\varphi_{2,c}+1}{2q_{C}}\right)\\ -\sum_{i=1}^{\varphi_{2,c1}}\frac{i}{q_{A}-q_{r2}}-\sum_{i=\varphi_{2,c1}+1}^{\varphi_{2,c1}+\varphi_{2,c2}}\frac{i-\varphi_{2,c1}}{q_{A}-q_{r1}}-\sum_{i=\varphi_{2,c1}+\varphi_{2,c2}+1}^{\varphi_{2,c}}\frac{i-\varphi_{2,c1}-\varphi_{2,c2}}{q_{A}-q_{r2}} \end{array}\right) \end{array}$$ (24f)

Above all, we can draw a conclusion of the expected value of the average travel delay in the case of *N* = 2, which is given by

$$E\left(D_{1|2}\right)=E\left(D_{1|2,\bar{B}}\right)P_{1}\left(\bar{B}\right)+E\left(D_{1|2,a}\right)P_{1}\left(B_{a}\right)+E\left(D_{1|2,c}\right)P_{1}\left(B_{c}\right)$$ (25)

Where the approximate formulas of $E\left(D_{1|2,\bar{B}}\right)$, $P_{1}\left(\bar{B}\right)$, *E*(*D*_1|2,*a*_), *P*_1_(*B*_*a*_), *E*(*D*_1|2,*c*_) and *P*_1_(*B*_*c*_) are Eqs 19, 18d, 20e, 21, 24f and 22, respectively.

### Effect of MBs with multiple different velocities

We recognize that in cases of MBs with multiple different velocities, when *N* ≥ 3, one can identify “embedded renewal processes” [22] that can be solved sequentially in order to get analytical formula for average travel delay.

With the view of mathematical tractability, we take the array between two successive MB_*N*-2_s with MB_*N*-1_s and MB_*N*_s inside only as a new MB^*N*-1^ (otherwise a new renewal takes place and the process starts over). With regard to these new MBs, the average travel delay can be obtained by Eq 25. Notice that, unlike the case of MBs with two different velocities, values of *p*_*SV*(*N*-1)_ and *p*_*SVN*_ are computed by the proportion of SVs with velocity of *v*_*Bj*_, where the sum of all *p*_*SVj*_, *j* = 1…*N*, equals 1. Then, we repeat this recursion to take the array between two successive MB_*N*-3_s with MB_*N*-2_s and the new MB^*N*-1^s inside only as a new MB^*N*-2^. Also, we use Eq 25 to solve the problem, in which replace *p*_*SV*1_ with the proportion of MB_*N*-2_, namely *p*_*SV*(*N*-2),_ and *p*_*SV*2_ with the proportion of the new MB^*N*-2^, namely $\sum_{k=N-1}^{N}p_{SVk}=G\left(v_{BN}\right)-G\left(v_{B\left(N-2\right)}\right)$. Similarly, we can repeat the recursion by solving a series of two different velocities problems until the outermost layer of recursion with MB_1_s and the new MB^2^s, whose solution is analogous to Eq 25. This can be accomplished by solving the following recursion for *j* = *N*-1,…,1 with *E*(*D*_*N|N+*1_) = *E*(*D*_*N*_) and *G*(*v*_*B*0_) = 0. To generalized approximate formulas of related variables in the former section, one just needs to replace subscript 1 by *j* and 2 by (*j*+1).

$$E\left(D_{j|j+1}\right)=E\left(D_{j|j+1,\bar{B}}\right)P_{j}\left(\bar{B}\right)+E\left(D_{j|j+1,a}\right)P_{j}\left(B_{a}\right)+E\left(D_{j|j+1,c}\right)P_{j}\left(B_{c}\right)$$ (26a)

$$E\left(D_{j|j+1,\bar{B}}\right)=\frac{p_{SVj}}{\sum_{k=j}^{N}p_{SVk}}E\left(D_{j}\right)+\left(1-\frac{p_{SVj}}{\sum_{k=j}^{N}p_{SVk}}\right)E\left(D_{j+1|j+2}\right)$$ (26b)

$$P_{j}\left(A\right)=1-e^{-p_{SVj}\lambda_{SV}\tau_{upj}},P_{j}\left(C\right)=1-e^{-\sum_{k=j}^{N}p_{SVk}\lambda_{SV}\tau_{upj}},P_{j}\left(B\right)=e^{-p_{SVj}\lambda_{SV}\tau_{upj}}-e^{-\sum_{k=j}^{N}p_{SVk}\lambda_{SV}\tau_{upj}}$$ (26c)

$$P_{j}\left(\bar{B}\right)=1+e^{-\sum_{k=j}^{N}p_{SVk}\lambda_{SV}\tau_{upj}}-e^{-p_{SVj}\lambda_{SV}\tau_{upj}}$$ (26d)

$$E\left(D_{j|j+1,a}\right)=\frac{1}{q_{A}\left(E\left(\tilde{h}\right){|}_{{\tilde{h}}_{j|j+1}^{*}}^{\tau_{upj}}+\tau_{up(j+1)}\right)}\left(\begin{array}{l} \varphi_{j,a}L\left(\frac{1}{v_{Bj}}-\frac{1}{v_{A}}\right)+\frac{\varphi_{j,a}\left(\varphi_{j,a}+1\right)}{2}\left(\frac{1}{q_{C}}-\frac{1}{q_{A}-q_{rj}}\right)\\ +\varphi_{j+1,a}L\left(\frac{1}{v_{B(j+1)}}-\frac{1}{v_{A}}\right)+\frac{\varphi_{j+1,a}\left(\varphi_{j+1,a}+1\right)}{2}\left(\frac{1}{q_{C}}-\frac{1}{q_{A}-q_{r(j+1)}}\right) \end{array}\right)$$ (26e)

$$P_{j}\left(B_{a}\right)=\left(1-e^{-\sum_{k=j}^{N}p_{SVk}\lambda_{SV}\tau_{upj}}\right)e^{-\sum_{k=j}^{N}p_{SVk}\lambda_{SV}{\tilde{h}}_{j|j+1}^{*}}-\left(1-e^{-p_{SVj}\lambda_{SV}\tau_{upj}}\right)e^{-p_{SVj}\lambda_{SV}{\tilde{h}}_{j|j+1}^{*}}$$ (26f)

$$\begin{array}{c} E\left(D_{j|j+1,c}\right)=\frac{1}{q_{A}\left(E\left(\tilde{h}\right){|}_{\hat{\tau }}^{{\tilde{h}}_{j|j+1}^{*}}+\Delta t_{j+1,1}+\Delta t_{j+1,2}+\Delta t_{j+1,3}\right)}\\ {}*\left(\begin{array}{l} \varphi_{j,c}L\left(\frac{1}{v_{Bj}}-\frac{1}{v_{A}}\right)+\frac{\varphi_{j,c}\left(\varphi_{j,c}+1\right)}{2}\left(\frac{1}{q_{C}}-\frac{1}{q_{A}-q_{rj}}\right)+\varphi_{j+1,c}\left(C_{j|j+1}+\frac{\varphi_{j+1,c}+1}{2q_{C}}\right)\\ -\sum_{i=1}^{\varphi_{j+1,c1}}\frac{i}{q_{A}-q_{r(j+1)}}-\sum_{i=\varphi_{j+1,c1}+1}^{\varphi_{j+1,c1}+\varphi_{j+1,c2}}\frac{i-\varphi_{j+1,c1}}{q_{A}-q_{rj}}-\sum_{i=\varphi_{j+1,c1}+\varphi_{j+1,c2}+1}^{\varphi_{j+1,c}}\frac{i-\varphi_{j+1,c1}-\varphi_{j+1,c2}}{q_{A}-q_{r(j+1)}} \end{array}\right) \end{array}$$ (26g)

$$P_{j}\left(B_{c}\right)=P_{j}\left(B\right)-P_{j}\left(B_{a}\right)$$ (26h)

The recursion process can be simplified as

$$E\left(D_{j|j+1}\right)=a_{j}+b_{j}E\left(D_{j+1|j+2}\right)$$ (27a)

Where *a*_*j*_ and *b*_*j*_ are variables introduced for the purpose of simplifying the analytical expression, and the formulas of them are given by

$$a_{j}=\frac{p_{SVj}E\left(D_{j}\right)P_{j}\left(\bar{B}\right)}{G\left(v_{BN}\right)-G\left(v_{B\left(j-1\right)}\right)}+E\left(D_{j|j+1,a}\right)P_{j}\left(B_{a}\right)+E\left(D_{j|j+1,c}\right)P_{j}\left(B_{c}\right)$$ (27b)

$$b_{j}=P_{j}\left(\bar{B}\right)\left(1-\frac{p_{SVj}}{G\left(v_{BN}\right)-G\left(v_{B\left(j-1\right)}\right)}\right)$$ (27c)

It is straight forward to show that the average travel delay of vehicles influenced by MBs with multiple different velocities is approximately formulized to be

$$E\left(D\right)=E\left(D_{1|2}\right)=a_{1}+\sum_{j=2}^{N-1}a_{j}\prod_{k=1}^{j-1}b_{k}+E\left(D_{N}\right)\prod_{k=1}^{N-1}b_{k}$$ (28)

## Model validation and parameters sensitivity analysis

To check the validity of the proposed model, a series of input data should be obtained through field data. Open source database [23]of US highway 10 was used for our study because of its fine level of detail. This database includes 30-second counts and 5-minute records of flow, density, speed and volume, updated daily. We chose a basic segment with two lanes and four loop-detector stations from S980 to S983 in one direction. On account of the basic assumption that the initial traffic state remains stable, we considered the traffic stream from 9 a.m. to noon when the traffic stream maintains the flat peak state only, see in S2 and S3 Files.

### Input data preparation

**Fig 8:**
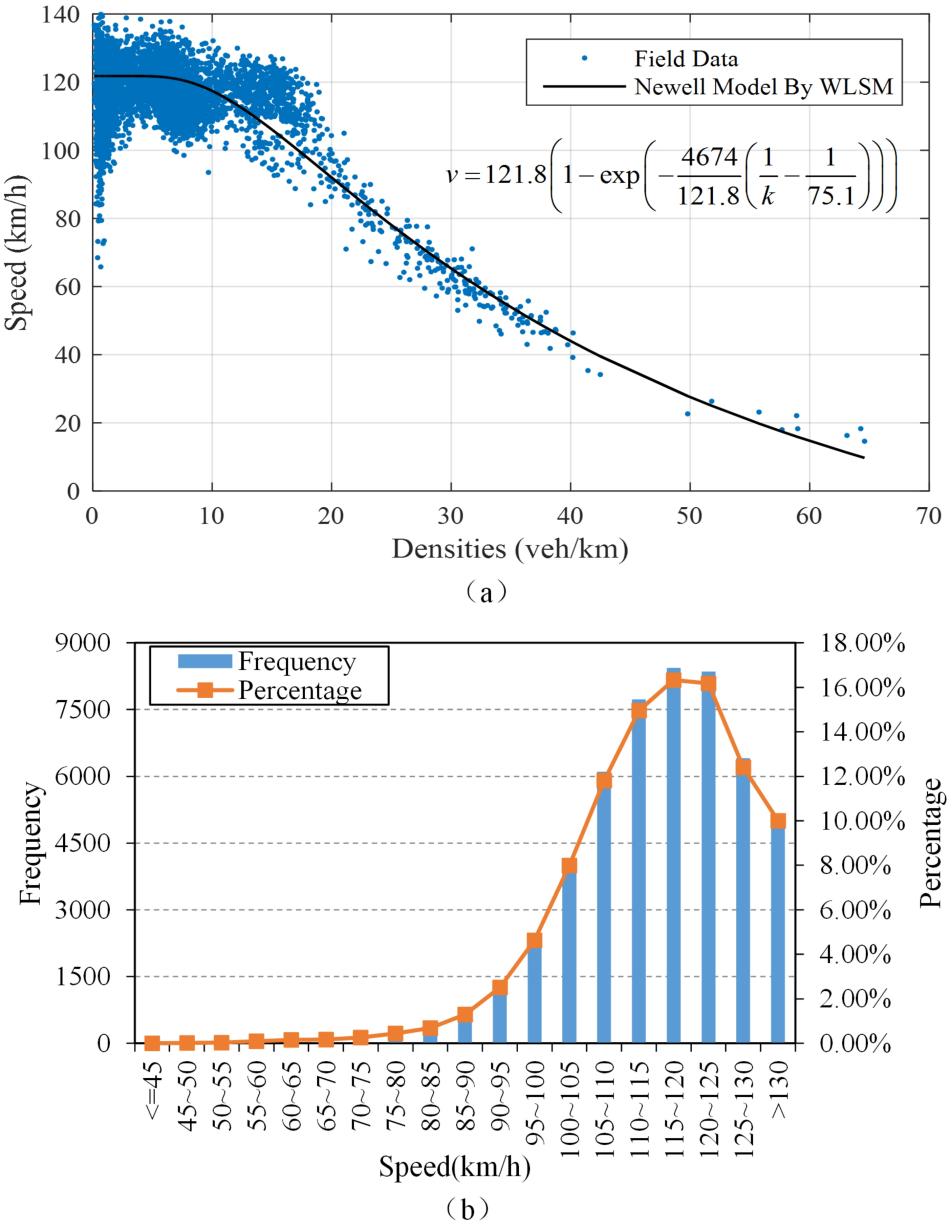
(a) Speed-density relationship: Field data vs. calibrated Newell model by WLSM; (b) frequency distribution histogram of speed.

At the very beginning, the speed-density or flow-density relationship which is the foundation of traffic flow theory needs to be acquired as the basic input data for our model. Due to the tremendous difference of the number of field data in different traffic state (the vast majority of those data were collected at light-traffic/free-flow conditions), existing single-regime models calibrated by the least square method (LSM) could not fit the empirical data consistently well both in light-traffic/free-flow conditions (the parameters calibrated using LSM are likely to be dominated by light-traffic/free-flow conditions). A weighted least square method (WLSM) proposed in [24] has solved this problem. Therefore, we fit the speed-density relationship in Newell model by WLSM, shown as Fig 8(a). Based on this well calibrated Newell model, it is straight forward to capture the three principal macroscopic parameters needed for each traffic state. The capacity of this basic segment is 1967veh/h with a corresponding travel speed of 70.5km/h.

To obtain the average travel speed of traffic state *A*, the frequency distribution of speed was analyzed with the result of Fig 8(b). The average speed ranged between 115.8km/h and 116.1km/h with 99% level of confidence. Hence, we adopted the only integer 116km/h as the speed of state *A*. The flow of state *A* was computed to be 1252veh/h.

Here we assumed that the desired speeds of SVs were restricted to a set of five values every 5km/h from 45km/h to 65km/h sharing the same proportion of 20% with *p*_*SV*_ = 1.0%. The corresponding flows were computed to be 1776veh/h, 1846veh/h, 1899veh/h, 1936veh/h and 1958veh/h, respectively. All of these *v*_*Bj*_ satisfied *q*_*rj*_ computed by Eq 3 smaller than *λ*_*qj*_ computed by Eq 4, which implied that all these SVs would cause queue propagating towards upstream. Based on this assumption, the case turned out to be MBs with 5 different velocities.

As for the critical gap *Γ* and follow-up time *η*, considerable researches had been down in the field of lane changing and gap acceptance studying. In the aspect of critical gap researching, Daganzo [25] used exponential, gamma, lognormal, normal, and normal distribution, respectively, to capture the variation seen in critical gaps and the conclusive value was 6s. Toledo et al.[26] found that the mean lane changing duration equals 4.6s. Later Moridpour et al.[27] concluded that for passenger cars the mean duration of a lane change equals 4.8s, whereas for trucks that equals 8.0s. Even though Kim et al.[28] took the relative speed into consideration for his Lane-changing gap acceptance model, the quantitative relationship between the critical gap and the relative speed remained unknown. Here we adopted the critical gap to be 5.4s, 5.2s, 5.0s, 4.8s and 4.6s for *v*_*B*1_ to *v*_*B*5_, respectively. These values were in the range of previous study results and conformed to the logic that the higher relative speed needs the larger critical gap. Similarly, in the aspect of follow-up time researching, Qu et al.[29] analyzed the follow-up time at a single-lane roundabout and the means of two groups of field data (first queuing vehicle or not) were 2.741s vs. 2.775s, of which the difference was not significant. The follow-up time was considered to be a saturation headway in a method for treating the traditional gap acceptance modelling by Akçelik [30] and a case of a four-lane uninterrupted major stream given by him [31] adopted *Γ = 6s* and *η = 3*.*6s*, while a general rule of thumb was *η/Γ* = 0.6 [32]. For overall consideration, we adopted the follow-up time to be 3.1s, 3.0s, 2.9s, 2.8s and 2.7s for v_B1_ to v_B5_. Notice that, these values were approximate and further works were required for values with better accuracy.

### Model validation

As presented in the part of introduction, most attention attracted by MBs was paid to its characteristics and impacts on capacity. There exists few previous research result that can be used as a comparison. The average travel delay was ever computed by Daganzo and Muñoz [4] through the actual travel time of vehicles in queue caused by a MB minus the time traveling with prevailing speed. Nevertheless, the delay was magnified due to the regardless of those passing vehicles. Also, the cross-sectional data collected by existing detectors cannot be used in this study due to the deficient density of detectors distribution. What is more, among all researches on MBs, only Muñoz and Daganzo [1] developed their research based on field observations and controlled experiments. All above prompted us to draw support from one of the commonly used traffic microscopic simulation tool VISSIM which allows users to model detailed geometric configurations as well as drivers’ behavioral characteristics encountered in the transportation system.

**Fig 9:**
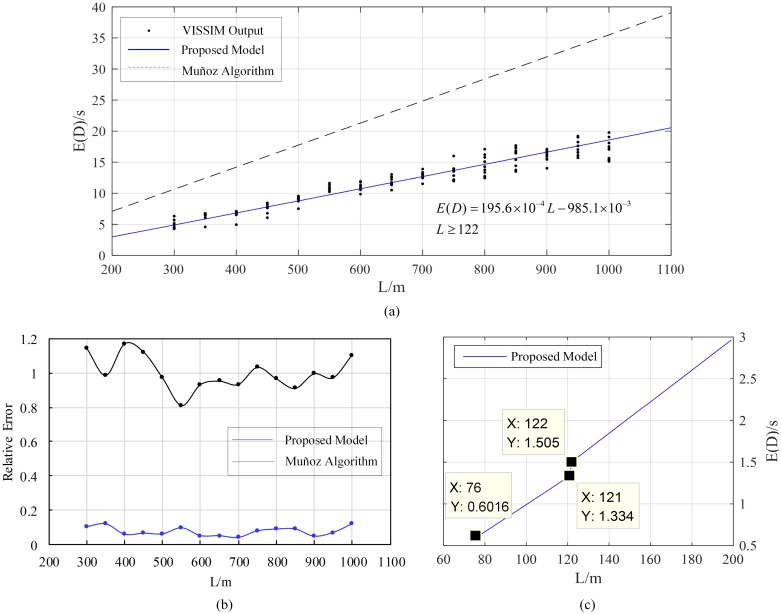
(a) E(D) vs L by results of VISSIM simulation and solution of proposed model; (b) relative errors of proposed model and Muñoz algorithm; (c) remarkable points in proposed model.

First of all, we used the prepared input data to calibrate the desired speed distribution of state *A* and SVs, the parameters in Wiedemann-99 driver behavior model, CC0 and CC1, which had been proved to have direct influence on capacity and are able to be determined in field [33,34]. Afterwards, multi-run simulation was done for a series of *L*. Delay data of vehicles influenced by MBs were abstracted and presented in Fig 9(a) together with the corresponding results of proposed model and Muñoz algorithm [4]. Notice that, when *L* was less than 76m, E(D) computed by the model equaled zero due to all *τ*_*upj*_ were not larger than the saturation headway, and when L was larger than 122m, all *τ*_*upj*_ were larger than it so that all SVs would become MBs, as shown in Fig 9(c). The relative errors of two methods compared with VISSIM output were shown in Fig 9(b). It is obvious that the traditional model used in Muñoz algorithm shows poor accuracy to predict all influenced vehicles’ travel delay regardless of passing vehicles while the proposed model shows considerable validity with good accuracy.

### Parameters sensitivity analysis

**Fig 10:**
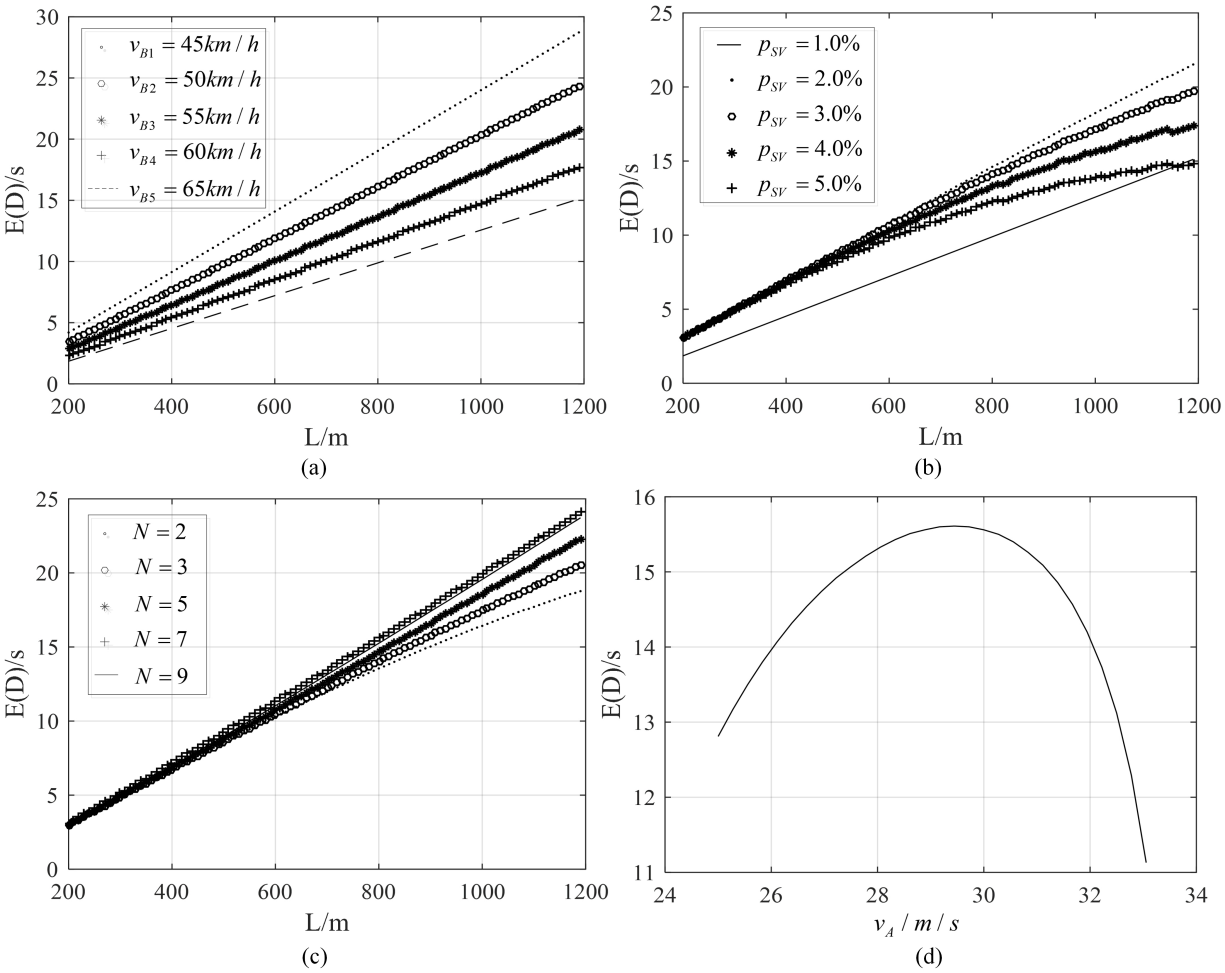
(a) Effect of *v*_*B*_ on *E*(*D*)-*L* relationship; (b) effect of proportion of SVs in traffic stream on *E*(*D*)-*L* relationship; (c) effect of N on *E*(*D*)-*L* relationship, and (d) effect of *v*_*A*_ on *E*(*D*) for *L* = 800m.

There are twelve input parameters in total for proposed model (see Table A in S1 File). Some of them are correlative. Here we select four independent parameters to analyze sensitivity. The results are illustrated in Fig 10. Fig 10(a) describes the average delay caused by MBs with a single velocity when *p*_*SV*_ = 1.0%. Different velocity results in different slope for linear relationship between *E*(*D*) and *L*. However, that relationship trends to nonlinear with the total proportion of SVs increasing, shown in Fig 10(b). The explanation for this phenomenon can be found in Eq 18c. As a lager *p*_*SV*_ results in a larger occurring probability of events set B which has *L* contained in the exponential variable *τ*_*up*_. For the range between 45km/h and 65km/h, the total number of different velocities *N* seems to show less difference when *N* is lager, shown in Fig 10(c). The main effect of *N* on that relationship is its contribution to the proportions of low velocities, which has been revealed by Laval [9]. The velocity of initial traffic state shows a relationship of analogous parabola with *E*(*D*). The peak value of *E*(*D*) appears at *v*_*A*_ = 29.4m/s when *L* = 800m, shown in Fig 10(d), and the value of *E*(*D*) increases with *v*_*A*_ when it is below 29.4m/s while decreases when lager than that value, which are caused by the increased flow and decreased initial travel speed respectively. Therefore, the flow of initial traffic state when the peak delay appears can be evaluated by the proposed model, based on which traffic management scheme can be proposed to restrict SVs from speed, proportion or travel time period to prevent delay beyond acceptance from occurring.

## Discussion and outlook

This paper derived formulas for the expected average travel delay of all vehicles influenced by MBs through a basic highway segment based on KW-MB theory, gap acceptance theory, probability theory and renewal theory. If one obtained the values of twelve input variables which were easy to achieve from field data, the expected average travel delay would be approximately formulated and computed, relying on which the related traffic management decisions could be made more scientifically. For example, based on the effect of ***v***_***B***_ on *E(D)*-*L* relationship, one can decide the minimum speed restriction of a special *L* by matching an acceptable range of *E(D)*; based on the effect of *v*_*A*_ on *E*(*D*) for a special *L*, one can obtain the range of *v*_*A*_ by matching an acceptable range of *E*(*D*), and afterwards, truck prohibited period decision could be made by matching that range with the speed-time distribution curve during a day; if the minimum speed restriction and prohibited period are invalid in case the minimum speed is larger than the full loaded speed of most types of truck or prohibited period spans all day long, truck lane restriction is a recommended traffic management strategy to reduce or even avoid MBs.

Though the problem stated in this paper is under a Poisson arrival process for SVs, the analytical method and modeling framework are generic by just substituting the related closed-form expression for certain variables. Moreover, several other parameters can be similarly approximately formulated in virtue of the modeling framework in this paper, such as the desired space-mean speed, real-time or maximum queuing length, and traveling tracks of those vehicles in queue.

During the derivation process, two main assumptions in previous research are followed: (i) the assumption that there exist SVs with a set of *N* discrete velocities, and (ii) the assumption that a faster SV will queue behind a slower one and is forced to travel at the same lower speed until the queue discharged. Actually, velocity of SVs follows a continuous distribution. But the difference has been testified insignificant in [9], and lines for N≥7 trend to coincide in Fig 10(c). Therefore this assumption is reasonable.

In reality, a certain proportion of those faster SVs would prefer to change lanes to avoid queuing. As a consequence, MBs will affect the traffic stream in more than one lane with interactions. However, lane-changing behavior of SVs has neither been taken into consideration in previous researches nor this paper due to far more complexity. Not only is a model for lane-changing behavior needed, but also the critical condition of a lane-changing for the faster SV queuing upstream of a slower SV. Accordingly, the following research work has been conducted by taking the traffic state in adjacent lane upstream of a slower SV as state *A* for the faster SV queuing upstream of it, with the aim of generalizing proposed model to multi-lane cases.

## Supporting information

S1 File Notations used during the derivation of approximate formulas. (PDF)

S2 File Raw data of 30-second interval downloaded from the open source database of US highway 10. (XLSX)

S3 File Raw data of 5-minute interval downloaded from the the open source database of US highway 10. (XLSX)
