# Supplementary material for: Evaluation of average travel delay caused by moving bottlenecks on highways
Source: PLoS One. 2017 Aug 30;12(8):e0183442. doi: 10.1371/journal.pone.0183442 (PMC5576711; doi:10.1371/journal.pone.0183442)
Supplement: S1 File — (PDF) [file pone.0183442.s001.pdf]

Table A Notations used during the derivation of approximate formulas

Symbol	Variables	Value
L	Travel distance of SVs caused MB-effect (m)	Input
q_A	Flow rate of the initial traffic state A (veh/s)	Input
v_A	Travel velocity of state A (m/s)	Input
q_C	Flow rate of the capacity traffic state C (veh/s)	Input
v_C	Travel velocity of state C (m/s)	Input
N	Number of different velocities (dimensionless)	Input
p_{SV}	Proportion of SVs in traffic stream (%)	Input
v_{Bj}	Travel velocity of SVs with a sequence number j (m/s)	Input
q_{Bj}	Flow rate of state B_j with a corresponding travel velocity v_{Bj} (veh/s)	Input
p_{SVj}	Proportion of SVs with travel velocity v_{Bj} in all SVs (%)	Input
Γ_j	Critical gap for vehicles influenced by MB$_j$ (s)	Input
η_j	Follow-up time for vehicles influenced by MB$_j$ (s)	Input
k_A	Density of the initial traffic state A (veh/m)	q_A/v_A
k_{Bj}	Density of state B_j with a corresponding travel velocity v_{Bj} (veh/m)	q_{Bj}/v_{Bj}
k_C	Density of the capacity traffic state C (veh/m)	q_C/v_C
w_{AB_j}	Propagation velocity of traffic shock wave caused by the transition between traffic state A and B_j (m/s)	$\frac{q_A - q_{Bj}}{k_A - k_{Bj}}$
w_{B_jC}	Propagation velocity of traffic shock wave caused by the	$\frac{q_{Bj} - q_C}{k_{Bj} - k_C}$

Symbol	Variables	Value
	transition between traffic state B_j and C (m/s)	
w_{AC}	Propagation velocity of traffic shock wave caused by the transition between traffic state A and C (m/s)	$\frac{q_A - q_C}{k_A - k_C}$
λ_A	Arrival rate of vehicles in state A (veh/s)	q_A
λ_{SV}	Arrival rate of SVs (veh/s)	$p_{SV}q_A$
$G(v_{Bj})$	Cumulative distribution function of v_{Bj} (%)	$\sum_{k=1}^{j} p_{SVk}$
$\hat{\tau}$	The minimum safety headway of those SVs (s)	$1/q_C$
$\tilde{h}$	Headway between SVs (s)	——
$E(m_j)$	Expected value of admissible changing-lane vehicles' number using a headway in left lane influenced by a MB_j (dimensionless)	Eq. (2)
q_{rj}	Passing rate of vehicles influenced by a MB_j (veh/s)	Eq. (17h)
ω_j	Propagation velocity of queuing shock wave caused by MBs taking passing rate into consideration (m/s)	Eq. (17f)
τ_{upj}	Disturbance time, during which vehicles in state A supposed to arrive at zero position will be influenced by MB_j (s)	Eq. (17g)
$I(t)$	Real-time length of queue upstream of a MB (m)	Eq. (8a)
T	The queue clearance time (s)	Eq. (8b)
t_{Lj}	Travel time of SV_js over a distance of L (s)	Eq. (8b)
t_{in}	Time at which a vehicle joins the queue caused by a MB (s)	Eq. (9b)
t_{out}	Time at which a vehicle discharges from the queue (s)	Eq. (9b)

Symbol	Variables	Value
t_i	Time at which a vehicle with a sequence number i of φ supposed to arrive at zero position and later on joining the queue without passing opportunities (s)	Eq. (9b、24b)
t_{Li}	Actual travel time of Vehicle i through distance L (s)	Eq. (12b、24a)
$\Xi\left(t_i,\tilde{h}\right)$	A 0-1 function whose return value only equals 0 or 1 based on a logical judgment (dimensionless)	Eq. (12c、13c)
$E(D_j)$	Expected value of average travel delay of all vehicles influenced by MB_js (s)	Eq. (17a)
D_j^i	Average travel delay of vehicles influenced by (i+1) MB_js (s)	Eq. (17b)
$F(D_j^i)$	Probability of D_j^i (%)	Eq. (17c)
φ_j^i	Total number of vehicles influenced by (i+1) MB_js and joining the queue without passing opportunities (dimensionless)	Eq. (17d)
$E\left(\tilde{h}\right)\vert_{\hat{\tau}}^{\tau_{upj}}$	Expected value of $\tilde{h}$ between two SV_js range from $\hat{\tau}$ to τ_{upj} (s)	Eq. (17e)
$\tilde{h}^*_{j\vert j+1}$	Critical headway between MB_j and MB_{j+1} to differentiate events set B_a and B_c (s)	Eq. (20a)
$E\left(\tilde{h}\right)\vert_{\tilde{h}^*_{j\vert j+1}}^{\tau_{upj}}$	Expected value of $\tilde{h}$ between MB_j and MB_{j+1} in events set B_a (s)	Eq. (20b)
$\varphi_{j,a}$	Number of vehicles influenced by MB_j in events set B_a	Eq. (20c)

Symbol	Variables	Value
	(dimensionless)	
$\varphi_{j+1,a}$	Number of vehicles influenced by MB_{j+1} in events set B_a (dimensionless)	Eq. (20d)
$E(\tilde{h})\|_{\hat{\tau}}^{\tilde{h}^*_{j\|j+1}}$	Expected value of $\tilde{h}$ between MB_j and MB_{j+1} in events set B_c (s)	Eq. (23)
$\varphi_{j+1,c1}$	Number of stage 1 vehicles influenced by MB_{j+1} in events set B_c (dimensionless)	Eq. (24c)
$\varphi_{j+1,c2}$	Number of stage 2 vehicles influenced by MB_{j+1} in events set B_c (dimensionless)	Eq. (24c)
$\varphi_{j+1,c3}$	Number of stage 3 vehicles influenced by MB_{j+1} in events set B_c (dimensionless)	Eq. (24c)
$\varphi_{j+1,c}$	Total number of vehicles influenced by MB_{j+1} in events set B_c (dimensionless)	Eq. (24c)
$\varphi_{j,c}$	Number of vehicles influenced by MB_j in events set B_c (dimensionless)	Eq. (24d)
$C_{j\|j+1}$	A constant introduced for the purpose of simplifying the formula (s)	Eq. (24e)
$E(D_{j\|j+1})$	Expected average travel delay of all vehicles influenced by MB_js and MB_{j+1}s (s)	Eq. (26a)
$E(D_{j\|j+1,\bar{B}})$	Expected average travel delay of all vehicles influenced by MB_js and MB_{j+1}s in events set $\overline{B}$ (s)	Eq. (26b)

Symbol	Variables	Value
$P_j(A)$	Probability of occurrence of events set A that there exists an interaction between two MB_j (%)	Eq. (26c)
$P_j(B)$	Probability of occurrence of events set B that there exists an interaction between a MB_j and a MB_{j+1} (%)	Eq. (26c)
$P_j(C)$	Probability of occurrence of events set C that there exists an interaction between a MB_j and another MB (%)	Eq. (26c)
$P_j(\bar{B})$	Probability of occurrence of complementary events set of events set B that there exists no interactions between a MB_j and a MB_{j+1} (%)	Eq. (26d)
$E\left(D_{j\|j+1,a}\right)$	Expected average travel delay of all vehicles influenced by MB_js and MB_{j+1}s in events set B_a (s)	Eq. (26e)
$P_j(B_a)$	Probability of occurrence of events set B_a for MB_js (%)	Eq. (26f)
$E\left(D_{j\|j+1,c}\right)$	Expected average travel delay of all vehicles influenced by MB_js and MB_{j+1}s in events set B_c (s)	Eq. (26g)
$P_j(B_c)$	Probability of occurrence of events set B_c for MB_js (%)	Eq. (26h)
a_j	A variable introduced for the purpose of simplifying the formula (s)	Eq. (27b)
b_j	A variable introduced for the purpose of simplifying the formula (%)	Eq. (27c)
$E(D)$	Expected average travel delay of all vehicles influenced by all MBs with different velocities (s)	Eq. (28)
